# Embodied Greenhouse Gas Emissions in Diets

**DOI:** 10.1371/journal.pone.0062228

**Published:** 2013-05-15

**Authors:** Prajal Pradhan, Dominik E. Reusser, Juergen P. Kropp

**Affiliations:** 1 Potsdam Institute for Climate Impact Research, Potsdam, Germany; 2 University of Potsdam, Dept. of Geo- and Environmental Sciences, Potsdam, Germany; DOE Pacific Northwest National Laboratory, United States of America

## Abstract

Changing food consumption patterns and associated greenhouse gas (GHG) emissions have been a matter of scientific debate for decades. The agricultural sector is one of the major GHG emitters and thus holds a large potential for climate change mitigation through optimal management and dietary changes. We assess this potential, project emissions, and investigate dietary patterns and their changes globally on a per country basis between 1961 and 2007. Sixteen representative and spatially differentiated patterns with a per capita calorie intake ranging from 1,870 to 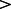
3,400 kcal/day were derived. Detailed analyses show that low calorie diets are decreasing worldwide, while in parallel diet composition is changing as well: a discernable shift towards more balanced diets in developing countries can be observed and steps towards more meat rich diets as a typical characteristics in developed countries. Low calorie diets which are mainly observable in developing countries show a similar emission burden than moderate and high calorie diets. This can be explained by a less efficient calorie production per unit of GHG emissions in developing countries. Very high calorie diets are common in the developed world and exhibit high total per capita emissions of 3.7–6.1 kg CO_2eq._/day due to high carbon intensity and high intake of animal products. In case of an unbridled demographic growth and changing dietary patterns the projected emissions from agriculture will approach 20 Gt CO_2eq._/yr by 2050.

## Introduction

Globally, food consumption patterns are changing both in terms of total amount and composition [1], [2]. Population growth and poverty reduction are commonly mentioned as major driving forces for this development which is expected to continue [1], [3]. Moreover, because of lifestyle related changes in diet compositions food demand will increase significantly, even with no further growth of global population [2], [4]. Food production usually requires inputs, like fuel and fertilizer [5], [6] and accounts for approx. 70% of global water withdrawal [7]. Current agricultural practices induce high environmental stress and in particular contribute 10–14% to the total anthropogenic greenhouse gas (GHG) emissions [8], [9]. Today agriculturally managed land covers about 38% of global land area [10], but inadequate agricultural practices have led to soil degradation in many regions in the past [11]. Concerning the fact that in some regions worldwide human attribution of net primary production approaches approx. 90% an array of additional problems in agriculture are foreseeable in the future [12], [13]. Consequently, during the last 50 years it has been observed that the agricultural land available to feed one person has been decreasing [4]. As a result of constraints on agricultural land availability the energy requirements for food production have increased [5]. Therefore, it is likely that the necessary increase in food production will exacerbate environmental stress and increase demand for external inputs [14].

A dietary shift towards a reduction in meat consumption has the potential to significantly decrease GHG emissions [5]–[20]. However, current trends are pointing in the opposite direction. Lifestyle related changes in diet with increased intake of animal products, vegetable oils and sugar-sweeteners had occurred mainly in Western Europe over the past few decades [21]. More recently, a westernization of diets has also been occurring in developing countries [22], [23]. Still, animal protein, animal fat and vegetable oil intake is significantly higher in developed countries compared to developing countries [24], [25].

To better understand diet related emissions, we identified typical dietary patterns on food consumption and composition per country by means of a self-organizing neural network approach for a database covering 1961–2007 [10], [26]. Based on these patterns we estimate GHG emissions embodied in the diets considering both agricultural non- CO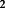 emissions and emissions related to fossil fuel use. To project future dietary patterns and associated total agricultural GHG emissions we use the relationships between diet and the development level of countries. Following the call to go beyond GDP [27], we measured development in terms of HDI values, while previous studies used per capita income [18], [23]–[25]. We estimate emissions for three scenarios: a) population growth only, b) population growth and changes in dietary patterns and c) change in population, diet as well as technology and management.

## Results

### Typical Patterns of Food Consumption

**Figure 1:**
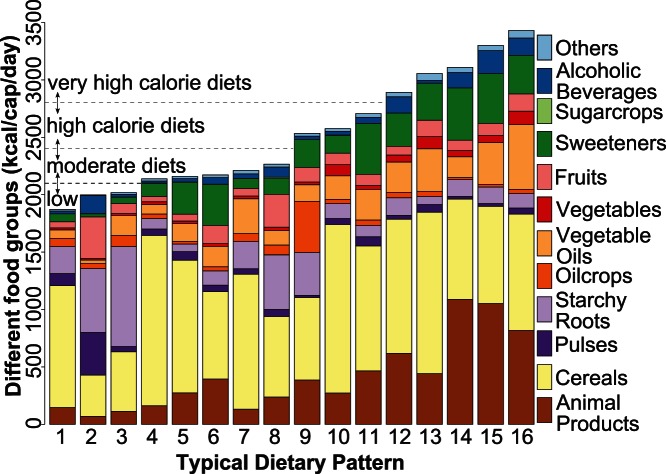
The sixteen dietary patterns observed world-wide for the analyzed period 1961–2007. “Others” represents the difference between the sum of all food groups and the total calorie intake. The sixteen identified patterns are categorized into low (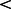
2,100 kcal/cap/day), moderate (2,100–2,400 kcal/cap/day), high (2,400–2,800 kcal/cap/day), and very high calorie diets (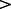
2,800 kcal/cap/day).

Global food consumption patterns can be represented by sixteen systematically derived dietary patterns. The patterns differ in regard to the food composition and energy content (Figure 1). For a plausible and brief discussion, we group the patterns into four groups related to the energy content, i.e. low (pattern #1– #3), moderate (pattern #4– #8), high (pattern #9– #11), and very high (pattern #12– #16) calorie diets (for a more encompassing description cf. Text S1).

The diets with low energy content provide less than 2,100 kcal/cap/day and are composed by more than 50% cereals (pattern #1) or more than 70% starchy roots, cereals, and pulses (pattern #3). Animal products play a minor role in this group (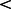
10%). All countries exhibiting low calorie diets are developing countries, mainly located in Africa and Asia. China was also a member of this group until 1977. Diets with a moderate energy content are characterized by 2,100–2,400 kcal/cap/day. One prominent example, namely pattern #4, is characterized by the fact that more than 70% of the energy is supplied by cereals. This prototype which was mostly observed in Africa and Asia represents the cultural habit of the usage of rice based diets. Countries belonging to the group of high calorie diets (2,400–2,800 kcal/cap/day) show particular regional characteristics, i.e. with a high fraction of fruits and oil crops (e.g. pattern #9). This archetype was mainly observed in Caribbean and Pacific island states. Very high calorie diets provide more than 2,800 kcal/cap/day. For these diets a high amount of meat and alcoholic beverages is representative (e.g. pattern #14 and #15). A Mediterranean style diet (pattern #16) is composed by a high fraction of vegetable oil, vegetables and fruits. This pattern is also prominent in Canada (since 1995) and the USA (since 2000) implying that a transition in food consumption may occur. Nevertheless, even among the very high calorie diets we found a pattern (#13) which is based on a high fraction of cereals combined with a very low consumption of alcoholic beverages, as it is culturally forced for certain African and Asian countries.

**Figure 2:**
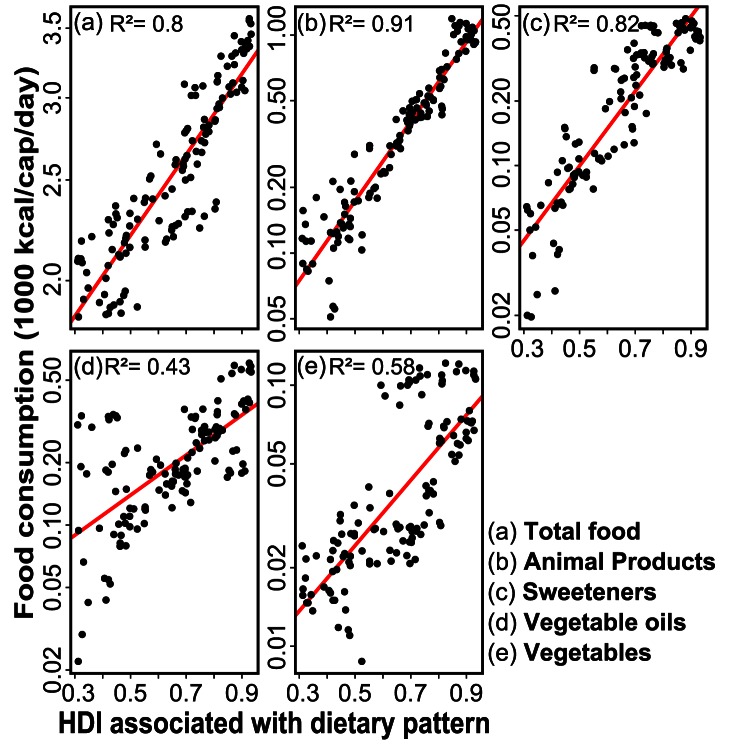
Relation between development (HDI) and consumption of total food (a), animal products (b), sweeteners (c), vegetable oils (d) and vegetables (e). Average values for each dietary pattern and year are shown. Note that the 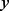-axis is in logarithmic scale, implying that a linear shape corresponds to an exponential relation.

Our examinations regarding the relation between dietary patterns and certain development levels show that the amount of total calories, animal products, sugar-sweeteners, vegetable oils and vegetables have a clear exponential relationship with the Human Development Index (HDI) (Figure 2).

### Transitions of Dietary Patterns

**Figure 3:**
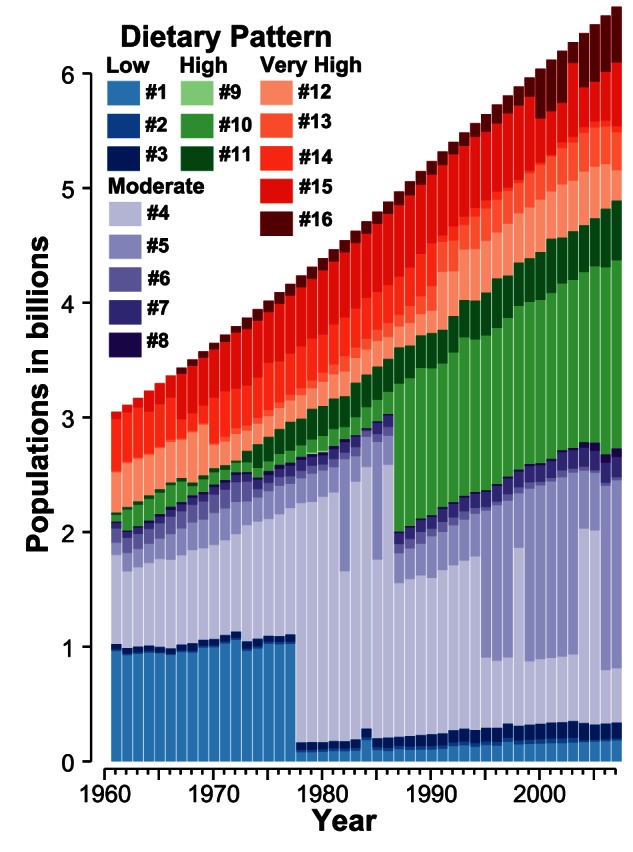
Number of people living on a certain dietary pattern for each year (1961–2007). The color codes represent the sixteen dietary patterns. The huge changes in the number of people consuming low calorie diets from 1977 to 1978 and on moderate calorie diets from 1986 to 1987 are due to dietary transitions in China. The huge fluctuation in the number of people consuming pattern #4 and pattern #5 diets (e.g. on 1982, 1985, 1995, etc.) is mainly because of shifting membership of India between pattern #4 and pattern #5. Note that pattern #16 newly emerged in the middle of the 1960ties.

In agreement with the fact that the long-term nutrition state is improving, food consumption patterns move from low towards higher calorie diets. Accordingly, the number of people living on low calorie diets is decreasing, while the number of people that are consuming high calorie diets is increasing (Figure 3). In particular the situation in the nourishment status of China changed from 1977 to 1978 (Figure 3, #1 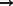 #4). After the end of the cultural revolution China started with drastic economic reforms leading to a much better food supply. Other fluctuations are related to a changing membership for India (#4 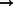 #5) (e.g. on 1982, 1985, 1995, etc.). In addition, the applied neural network approach also detected a newly emerging pattern in the middle of the 1960ties revealing that lifestyles are changing. This diet archetype (#16) is comparatively healthy, i.e. it is composed of a good share of vegetable oils, vegetables, and fruits. At the end of the analyzed period this consumption style comprised approx. 500 million people. Maps for different years showing the spatial distribution of the patterns are presented in Figure S1.

**Figure 4:**
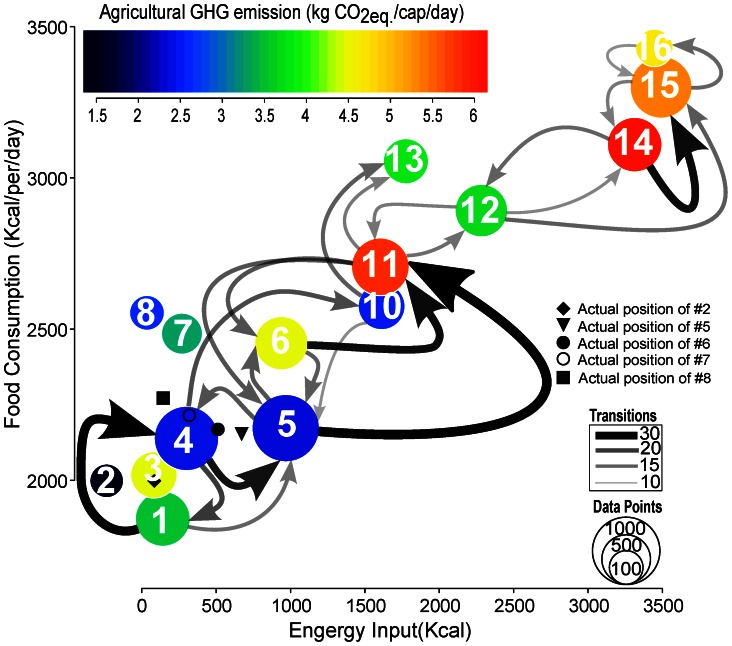
Graphical representation of the most characteristic transitions pathways. Arrows of varying thickness show the number of changes occurring for all countries over the entire period. Changes occurring fewer than ten times are omitted; for a tabular representation and further details cf. also Table 2. Note, that pattern #9 is not shown due to missing data about energy input. The diagram shows fossil energy input (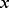-axis), total food consumption (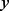-axis), total GHG emissions (color codes), pattern importance (as total number of country and year represented by the point size). For the description of diet typologies refer to Figure 1.

More detailed examinations identify typical transitions pathways (cf. Figure 4 and Table S1). The figure makes clear that globally a clear tendency exists for shifts from low towards high calorie diets, while in parallel the needed energy input is also increasing. All the shifts represent certain life-styles which are expressed in the diet composition. One of the most prominent shifts is that from pattern #1 to #4 which represents an increase in the total calorie intake, but retains cereals as the major energy source. A subsequent transition is that from #10 to #13. Overall this pathway corresponds to an increase in the calorie content associated with a fairly constant food composition. Countries who showed this change were mainly located in Asia and North Africa. A possible explanation is the limited affordability of animal products combined with local environmental conditions and religious taboos influencing meat and alcohol consumption [24]. Other frequent transitions are those from #6 to #11, #14 to #15, and #4 to #5, which are all characterized by changing food composition to an increased share of animal products, vegetable oils and sugar-sweeteners. This mostly occurred in European, American and some African countries. Examples are Brazil (#6 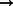 #11) in 1974, Germany (#14 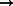 #15) in 1964 and for USA in 1967, and Kenya (#4 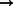 #5) in 1980. Further often observed pattern shifts, i.e. #5 to #11, represent an increase in mainly animal products, sweeteners and fruits, while retaining a relatively high fraction of pulses. This shift held for Mexico in 1973 and for Thailand in 2006. A trend which is mainly observable in industrialized countries is reflected by the shift from pattern #15 (high amount of animal products, sugar sweeteners and alcoholic beverages) to pattern #16 (comparatively more vegetables and fruits). This obviously represents a shift in the mind-set of the consumers, i.e. that reports on research findings on negative health consequences associated with a meat rich diet [28].

In face of the overarching trend towards higher calorie diets some exceptions exist. On the one hand, dietary patterns #2, #3, #7, and #8 remain constant over time and are largely disconnected from other trends. As a major reason no significant changes in the development status of some states in Middle, Central, Western, and Eastern African states were identified. On the other hand, the applied approach is able to reconstruct changes towards diets with a lower calorie content. Such a development can be often associated with conflicts and other societal disruptions. For example, during the civil war in Angola (1980–1987) the diet changed from diet typology #3 to #1. Similarly, during the collapse of the former Soviet Union and Yugoslavia in most of the succession states the calorie intake decreased and moved from #14 to #12 or even #12 to #10, respectively.

### Embodied Fossil Energy and GHG Emissions

Countries characterized by high calorie diets exhibit a production mode which needs high fossil energy inputs (1,800–3,500 kcal/cap/day). In other words almost 1 kcal of fossil energy per kcal consumed is required. In countries with low calorie diets, the energy input can be as low as 80–150 kcal/cap/day (Figure 4 and Table 1). Converting this to fossil fuel related GHG emissions we found a range between 0.64 and 1.35 kg CO_2eq._/cap/day for very high calorie diets and between 0.03 and 0.05 kg CO_2eq._/cap/day for low calorie diets. For consideration of the overall GHG emissions one needs to include non- CO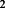 GHG emissions from enteric fermentation, rice cultivation, manure management and agricultural soils. These non- CO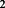 GHG emission intensities are in general relatively high for low and the moderate calorie diets (e.g. pattern #1, #3, #6 and #7) and result in high total emissions for these patterns (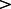
3 kg CO_2eq._/cap/day). In contrast, the non- CO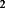 GHG emission intensities for crop and livestock are smaller for the very high and the high calorie diets group (Table 1). Thus, the high energy input and management strategies make agriculture more productive in developed as in developing countries [29], [30]. Thus, more agricultural goods can be harvested for the same amount of non- CO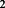 GHG emissions. At the same time, dietary shifts towards diets that include less animal products would have a great potential for climate change mitigation [15]–[19], [31], which is reflected in the higher non- CO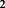 GHG emission intensity for livestock (1.44 to 13.06 g CO_2eq._/kcal) compared to crop production (0.31 to 1.81 g CO_2eq._/kcal). Consequently, the total GHG emissions are only slightly higher for high and very high calorie diets (2.48–6.10 kg CO_2eq._/cap/day) compared to low and moderate calorie diets (1.43–4.48 kg CO_2eq._/cap/day; Figure 4 and Table 1). Thus, in regard to the attribution of emissions to the agricultural sector, our approach provides a step forward by associating fossil fuel input and non- CO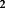 GHG emissions explicitly to different food consumption patterns.

**Table 1:** Environmental impact data for the different dietary patterns.

Pattern	Crop Products	Animal Products	Feed	Non- CO_2_ GHG Emissions		Energy O/I Ratio	Total Fossil Energy	Emissions from	Total GHG Emissions
	*PC_z_*	*PA_z_*	*F_z_*	Intensity (g CO_2eq._/kcal)		*R_O/Iz_*	*FE_z_*	Fossil Energy	*ET_z_*
	(kcal/cap/day)			Crop *ec_z_*	Livestock *ea_z_*		(kcal/cap/day)	(kg CO_2eq._/cap/day)	
1	1727	146	268	0.82	12.48	14.1	141.8	0.05	3.51
2	1928	71	297	0.42	6.58	26.2	85.0	0.03	1.43
3	1904	114	278	1.81	4.30	26.6	82.0	0.03	4.46
4	1977	161	408	0.64	4.75	7.9	301.2	0.11	2.39
5	1885	273	511	0.50	3.25	3.6	675.5	0.24	2.34
6	1778	394	820	1.07	3.83	5.0	521.2	0.19	4.48
7	2078	133	445	0.57	13.06	8.1	312.3	0.11	3.29
8	2028	238	665	0.44	5.59	20.3	133.0	0.05	2.56
9	2149	386	138	N/A	N/A	N/A	N/A	N/A	N/A
10	2303	272	1061	0.37	2.37	2.1	1612.4	0.58	2.48
11	2240	465	1364	0.91	4.25	2.3	1603.8	0.58	5.85
12	2273	619	2681	0.32	2.02	2.2	2282.0	0.83	3.68
13	2613	442	1762	0.52	1.96	2.5	1775.5	0.64	3.78
14	2020	1090	3662	0.51	1.84	1.7	3313.2	1.20	6.10
15	2248	1052	5037	0.32	1.70	2.1	3490.9	1.26	5.37
16	2611	818	4701	0.31	1.44	2.1	3445.1	1.35	4.66

Values are calculated based on equations 2–5 (Materials and Methods, and Text S2). No information on energy O/I ratio and agricultural GHG emissions was available for countries belonging to cluster 9. The table also reflects different agricultural practices. For example, for pattern #1 the high emissions are obviously related to an inefficient livestock management (cmp. column 6 and 10).

**Figure 5:**
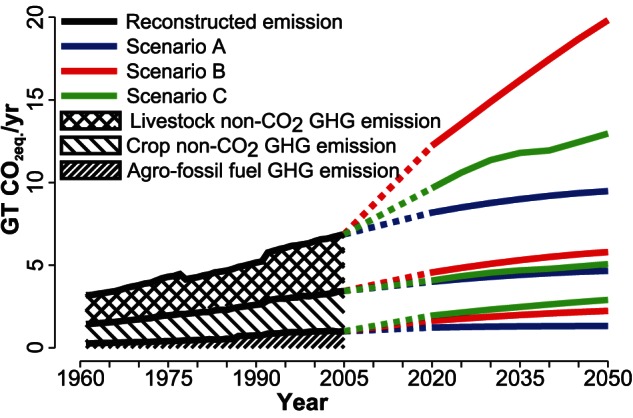
Reconstructed and projected global total agricultural GHG emissions for three scenarios. A: population growth only, B: population growth and changes in dietary patterns, C: change in population, diets as well as technology and management. Total GHG emissions are decomposed into non- CO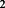 GHG emissions from livestock and crop, and GHG emissions from use of fossil fuel in agriculture. The depression in 1978 is mainly due to change in dietary pattern of China from pattern #1 to pattern #4 and the related lower non- CO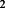 GHG emissions from livestock. The sharp rise in emissions in 1992 is caused by inclusion of the countries from the former Soviet Union in the analysis from 1992 onwards.

For future development Figure 5 shows that the agriculture related GHG emissions will increase by about 40% in 2050 compared to that in 2005 [9] due to population growth and hence increased food demand (Scenario A) (cf. also Figure S2). When changes in dietary patterns, i.e. lifestyle evolutions, are considered emissions will more than double to about 19.8 Gt CO_2eq._/yr in 2050 (Scenario B). Taking into account that for not transgressing the 2°C global warming target (25% probability of overshooting) an annual emission budget should be kept below 30 Gt CO_2eq._/yr [32]. This highlights the tremendous potential the food sector can play in regard to ambitious climate protection goals, while on the opposite the increase in food demand can be an essential component in future climate disruptions [2], [4]. If one consider improved agricultural practices (Scenario C) about 65% of non- CO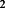 GHG emissions can be avoided through higher productivity. However, such modified agricultural practices in 2050 require about 33.6 EJ/yr energy input through fossil fuels, which is about 30% more than the fuel energy needed if the agricultural practices remain similar to 2007 (Figure S3) indicating that also this part can contribute considerably to emission reduction. The reduction potential of the livestock sector is shown in Figure 5. This sector is the major contributor of the agricultural non- CO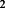 GHG emissions sharing more than 50%, 70% and 60% of the total GHG emissions in Scenarios A, B and C, respectively. In contrast, differences between the three scenarios are small for CO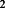 emissions from fossil fuels and non- CO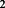 emissions from crops.

## Discussion

We present important changes of food consumption styles over the last 50 years for all countries with 16 distinct dietary patterns. We found that these patterns are typical for certain regions. They changed over time and in regard to the composition of diets as well as to the total calorie consumption. Environmental impacts in terms of fossil fuel requirements and the total GHG emissions generally increased as diets become more calorie rich.

With our approach we provide several innovations for the assessment of food consumption patterns. The advantage of the SOMTOP method (see Materials and Method) used for pattern recognition enables a non-linear dimensionality reduction and feature clustering. As an outcome a systematic data-driven archetypical model can be built which supplies valuable and systematic insights into the relations of features of the certain food patterns in comparison to empirical approaches implemented by others [33]. The SOMTOP is robust in the presence of non-linearities and assures that the topological structure of the data is maintained when mapping data from a high-dimensional onto a low-dimensional space in order to detect the degrees of freedom.

The spatial distribution of the patterns supports findings of previous studies, such as a high amount of consumed animal products in diets for North America, Europe and developed states in the Pacific [24]. This holds as well for developing countries in which a high proportion of cereals and starchy roots are important for the nourishment of the people. Nevertheless, such a comprehensive global analysis of existing dietary patterns has not been undertaken before. It was feasible to assess dietary patterns consistently for such long-term data and to derive a comprehensive understanding of changes in diets from these patterns. It was one advantage of the used method that it could detect newly emerging patterns during the course of time. Finally, the estimation of GHG emissions (fossil fuel based and non- CO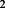 ) associated with these dietary patterns adds a further uniqueness to our study.

The findings contribute to the on-going discussion of the need for sustainable agricultural intensification [34]–[36]. It was shown that the non- CO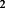 GHG emission intensities of developing countries are larger compared to developed countries (Table 1) and therefore, have great potential to contribute to emission reductions. Our scenarios suggest that optimized management may contribute to emission reductions of up to 7 Gt CO_2eq._/yr in 2050. Our approach also highlights the importance of the livestock sector for diet related GHG emissions. Emissions from this sector are increasing rapidly according to our estimations and about 14 Gt CO_2eq._/yr by 2050 (Scenario B) will be related to the consumption of animal products. Summing-up, agricultural intensification should focus on an optimization of emission intensities, while at the same time keeping other environmental stresses and anthropogenic inputs as low as possible. This will be addressed in a following study which will deal with the compatibility of our projections with current and future land-use demands.

## Materials and Methods

### Food Consumption Patterns

We characterized dietary patterns using global time series data on total food consumption and food composition per country from 1961 to 2007 from FAOSTAT [10]. The data cover 11 food categories, which contribute to more than 90% of the global food supply and the total food consumption in kcal/capita/day. The 11 food groups comprise animal products, cereals, pulses, starchy roots, oilcrops, vegetable oils, vegetables, fruits, sugar-sweeteners, sugarcrops and alcoholic beverages. The food data cover 217 countries and country groups e.g. Asia, Europe, World. The data comprise of 9 145 items (pairs of countries and years) made up of 12 input variables. A number of conventional multi-variate statistical methods can be used to identify patterns from the data, but they have some common limitations (e.g. the data is often considered normally distributed [37]; the variables are usually assumed to be correlated [38]; and the data is commonly expected to exhibit stationarity [39]). It was found that the food data set is not normally distributed and exhibits non-linearities between the variables. Additionally, due to changing food consumption patterns [1], [2], it is hard to expect stationarity in the data. Therefore, we used a self-organizing map (SOM) [26] to cluster the data, which is a robust method for non-linear and noisy data.

A SOM is a neural network that can be interpreted as a self-supervised clustering and non-linear dimensionality reduction technique. It also tries to preserve the topological ordering of the input data in the low-dimensional network space. Compared to other clustering (e.g. hierarchical and k-means) and linear dimension reduction approaches (e.g. PCA and MDS), the SOM creates a topologically ordered segmentation of the data space [40] and conceptualizes input vectors as representative samples from an 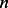-dimensional information continuum instead of discrete objects [41]. The self-organizing map was coupled with a neighborhood distortion measurement approach (-TOP) [42]. This part provides a quantitative measure 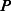 to estimate topological distortions during the classification process [42]. An optimal dimension is found when 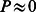, while 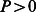 indicates a too large and 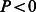 (Table 3) a too small dimension. Thus, the dietary patterns we identified are motivated by the structure in the data (cf. also Text S2 and Figure S5). The source code of the SOMTOP model was developed in C and successfully validated and applied to several other studies [43], [44]. We determined a network with 16 representative nodes and a three-dimensional configuration (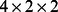) as optimal to describe the observed variation in the data sufficiently (about 72% of the variance explained, topographic product 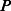 = 0.002
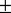
0.001, cf. Table 3). Each of the sixteen nodes represents a set of countries for different years having a certain food composition and total food supply. To derive diet transitions, the changes in dietary pattern of a country in consecutive years were considered.

### Relating Food Consumption to HDI

Data on the Human Development Index (HDI) from the HDI trend 1980–2007 [45] were used to determine the relationship between HDI and food consumption associated with the dietary patterns. Based on this relation and HDI projection taken from Costa et al. [46] we estimated future food consumption (Text S3).

### Assessing Fossil Energy and GHG Emissions

For estimating fossil energy and GHG emissions associated with the dietary patterns, we combined data on agricultural energy output/input (O/I) ratio (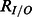) [5], on agricultural non- CO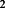 GHG emissions [8], on feed supply [10], on nutritive factors [47] and on food production [10]. First, we calculated the feed supply in kcal/cap/day (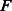) and the non- CO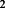 GHG emission intensity per kcal of crop products (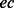) and animal products (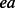) for each country from the FAOSTAT and agricultural non- CO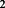 GHG emissions data (Text S2). To aggregate the impact data to the sixteen dietary patterns, we consider the sixteen sets (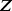) of pairs of countries (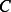) and years (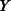), making up a certain dietary pattern, and related subsets 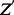 as the elements for which data on 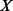 (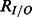, 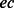, 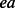 and 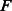) is available. Then the average value (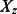) for a dietary pattern was calculated as the average from the set 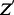 (equation 1). Subsequently, we will denote these dietary pattern specific averages as 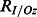 (energy O/I ratio), 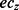 and 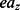 (non- CO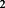 GHG emission intensities), and 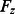 (feed use). From these values, we calculated GHG emissions from crop products (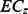), animal products (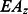) and total food consumption (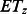) with equations 2, 3 and 4, respectively. 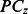 and 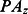 are the consumption of crop products (total food consumption minus animal products consumption) and animal products, respectively, while 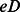 is the emission intensity of diesel (0.36 g CO_2eq._/kcal) [48], which was used to estimate GHG emissions from fossil energy. The required fossil energy input (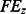) was estimated with equation 5. Note that equations 3 and 5 include a term to estimate environmental impacts from animal feed.
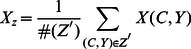
(1)

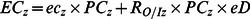
(2)

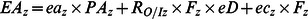
(3)

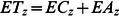
(4)

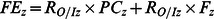
(5)


For future development, three scenarios were analyzed (Text S3): Scenario A considered population growth only; Scenario B represented population growth and changes in dietary patterns; and Scenario C contained changes in population, diets as well as technology and management. In order to project dietary patterns and estimate related GHG emissions from agriculture, we made use of the exponential relation between diet components and HDI (Figure 2) and combined it with HDI projections (cf. above).

## Supporting Information

Figure S1 **World-maps showing the spatiotemporal occurrence of the 16 dietary patterns.**
(PDF)

Figure S2 **Projected and reconstructed global food demand for three scenarios.** A: population growth only, B: population growth and changes in dietary patterns, C: change in population, diets as well as technology and management. Scenario B & C overlap because the only difference, agricultural technology and management do not affect food demand. The projected calorie demand (see Materials and Methods) was converted to the wheat equivalent using the nutritive factor of wheat.
(PDF)

Figure S3 **Projected and reconstructed global fossil fuel energy demand for the three scenarios (as Figure S2).**
(PDF)

Figure S4
**Projected and reconstructed global non- CO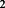 GHG emissions for the three scenarios (as Figure S2).** The figure also shows non- CO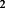 GHG emissions from US-EPA [8]. The reconstructed values slightly underestimate (less than 5%) the emissions by US-EPA. Reconstructed values are within the range of estimate presented by IPCC (5.1–6.1 Gt CO_2eq._/yr for year 2005) [9]. The projected emissions for the year 2050 (8.16 Gt CO_2eq._/yr, 17.58 Gt CO_2eq._/yr, and 10.06 Gt CO_2eq._/yr for Scenarios A,B, and C, respectively) are similar to values reported for scenarios from Popp et al. for 2055 (8.69 Gt CO_2eq._/yr for constant diet scenario on level of 1995, 15.3 Gt CO_2eq._/yr for increased meat scenario based on change in GDP and 9.78 Gt CO_2eq._/yr increased meat plus technological mitigation scenario) [18].(PDF)

Figure S5
**Schematic representation of a topology distortion.**(a) Representation of a topology distortion between two different network types. Consider the bullets as scatter plots in 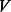. Such data distribution can be represented by a 1-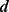 with 16 cells or by 3-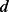 network with a 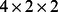 geometry (16 cells). While for the 1-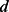 two bullets are direct neighbors in 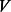, while in 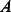 there have a maximum distance (black arrows). Only the 3-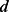 network can represent this topological ordering adequately (cf. Table S2 for the results of the actual simulations). (b) Measurement of the distances from point 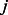 to the next neighbors of order one and two, if the points lying in 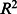 are mapped onto 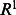. It is shown how distance ratios can be used in order to quantify topological distortions.(PDF)

Table S1
**Number of observed diet transitions. The counts on the diagonal represent unchanged dietary patterns.** Counts greater than 10 are used to plot the transition graph (Figure 4). Read from row to column, e.g. change recorded for Pattern 4 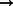 Pattern 5 is 27.(PDF)

Table S2
**Topographical product and data reconstruction rates for SOM maps of certain dimensions.**The map configuration with an optimal neighborhood preservation (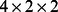) indicated by a topographical product 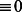 is used for further analysis (cf. arrow). It describes the 12-dimensional input manifold by a 3-dimensional embedding space and a data reconstruction rate of 70%.(PDF)

Text S1 **Characteristics of Identified Dietary Patterns.**
(PDF)

Text S2 **Methods and Data.**
(PDF)

Text S3 **Scenario Assumptions for the Estimation of GHGs from Agriculture.**
(PDF)
