# Correction: Embodied Greenhouse Gas Emissions in Diets

**DOI:** 10.1371/journal.pone.0159285

**Published:** 2016-07-08

**Authors:** Prajal Pradhan, Dominik E. Reusser, Juergen P. Kropp

There are errors in the Materials and Methods under the subheading “Assessing Fossil Energy and GHG Emissions.” The correct text is: For estimating fossil energy and GHG emissions associated with the dietary patterns, we combined data on agricultural energy output/input (O/I) ratio (*R*_*O/I*_) [5], on agricultural non-CO_2_ GHG emissions [8], on feed supply [10], on nutritive factors [47] and on food production [10]. First, we calculated the feed supply in kcal/cap/day (*F*) and the non-CO_2_ GHG emission intensity per kcal of crop products (*ec*) and animal products (*ea*) for each country from the FAOSTAT and agricultural non-CO_2_ GHG emissions data (S2 Text). To aggregate the impact data to the sixteen dietary patterns, we consider the sixteen sets (*Z*) of pairs of countries (*C*) and years (*Y*), making up a certain dietary pattern, and related subsets *Z'* as the elements for which data on *X* (*R*_*O/I*_, *ec*, *ea* and *F*) is available. Then the average value (*X*_*z*_) for a dietary pattern was calculated as the average from the set *Z'* (Eq 1). Subsequently, we will denote these dietary pattern specific averages as *R*_*O/Iz*_ (energy O/I ratio), *ec*_*z*_ and *ea*_*z*_ (non-CO_2_ GHG emission intensities), and *F*_*z*_ (feed use). From these values, we calculated GHG emissions from crop products (*EC*_*z*_), animal products (*EA*_*z*_) and total food consumption (*ET*_*z*_) with Eqs 2, 3 and 4, respectively. *PC*_*z*_ and *PA*_*z*_ are the consumption of crop products (total food consumption minus animal products consumption) and animal products, respectively, while *eD* is the emission intensity of diesel (0.36 g CO_2eq._/kcal) [48], which was used to estimate GHG emissions from fossil energy. The required fossil energy input (*FE*_*z*_) was estimated with Eq 5. Note that Eqs 3 and 5 include a term to estimate environmental impacts from animal feed.

$$X_{z}=\frac{1}{\#(Z\prime )}\sum_{(C,Y)\in Z\prime }X(C,Y)$$ (1)

$${EC}_{z}\;={ec}_{z}\times {PC}_{z}+\;\frac{{PC}_{z}}{R_{O/I_{z}}}\times eD$$ (2)

$${EA}_{z}\;={ea}_{z}\times {PA}_{z}+\;\frac{F_{z}}{R_{O/I_{z}}}\times eD+{ec}_{z}\times F_{z}$$ (3)

$${ET}_{z}\;={EC}_{z}+{EA}_{z}$$ (4)

$${FE}_{z}\;=\;\frac{{PC}_{z}+\;F_{z}}{R_{O/I_{z}}}$$ (5)

There are errors in the S2 Text under the heading “2.2 Data Sources and processing.” Please see the correct text here.

## Supporting Information

S2 Text Methods and Data. (DOCX)
