# Supplementary figures and images for: Embodied Greenhouse Gas Emissions in Diets
Source: PLoS One. 2013 May 15;8(5):e62228. doi: 10.1371/journal.pone.0062228 (PMC3655165; doi:10.1371/journal.pone.0062228)

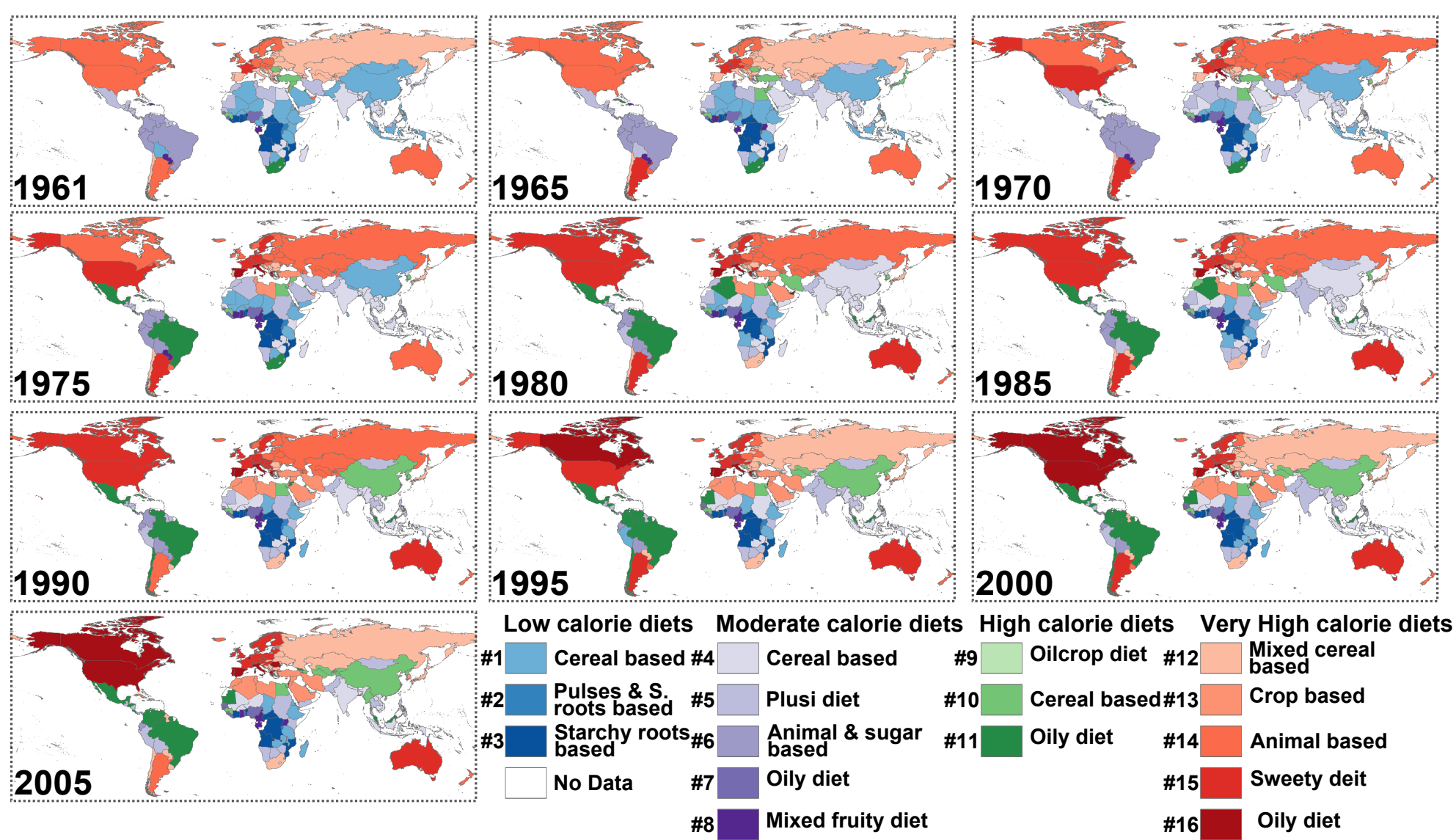

Supplement: Figure S1 — World-maps showing the spatiotemporal occurrence of the 16 dietary patterns. (PDF) [file pone.0062228.s001.pdf]

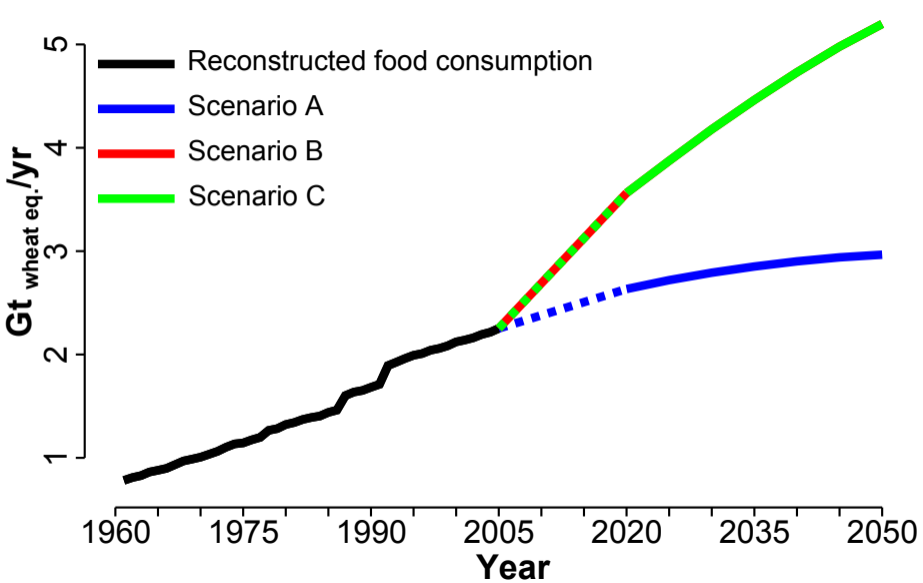

Supplement: Figure S2 — Projected and reconstructed global food demand for three scenarios. A: population growth only, B: population growth and changes in dietary patterns, C: change in population, diets as well as technology and management. Scenario B & C overlap because the only difference, agricultural technology and management do not affect food demand. The projected calorie demand (see Materials and Methods) was converted to the wheat equivalent using the nutritive factor of wheat. (PDF) [file pone.0062228.s002.pdf]

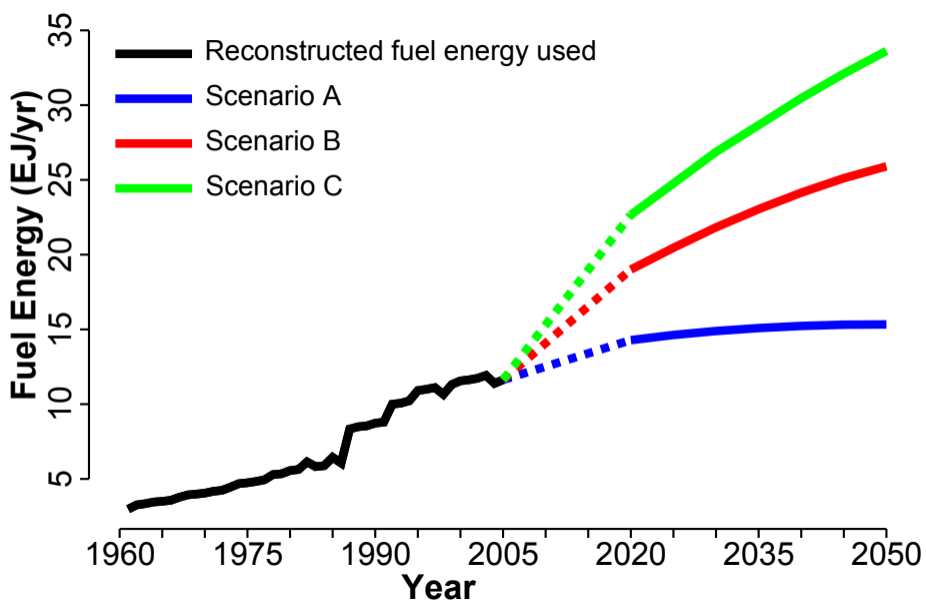

Supplement: Figure S3 — Projected and reconstructed global fossil fuel energy demand for the three scenarios (as Figure S2). (PDF) [file pone.0062228.s003.pdf]

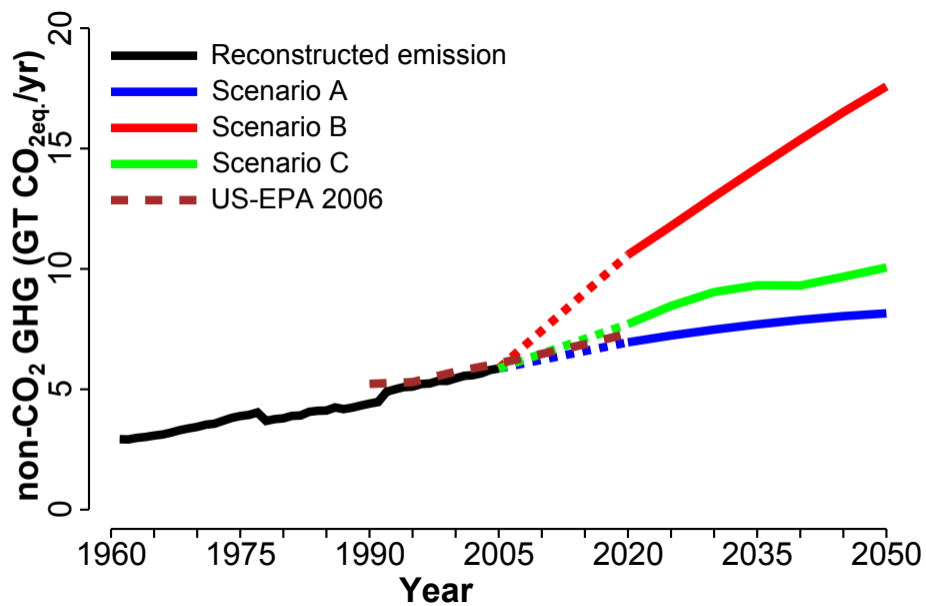

Supplement: Figure S4 — Projected and reconstructed global non- CO GHG emissions for the three scenarios (as Figure S2). The figure also shows non- CO GHG emissions from US-EPA [8]. The reconstructed values slightly underestimate (less than 5%) the emissions by US-EPA. Reconstructed values are within the range of estimate presented by IPCC (5.1–6.1 Gt CO2eq./yr for year 2005) [9]. The projected emissions for the year 2050 (8.16 Gt CO2eq./yr, 17.58 Gt CO2eq./yr, and 10.06 Gt CO2eq./yr for Scenarios A,B, and C, respectively) are similar to values reported for scenarios from Popp et al. for 2055 (8.69 Gt CO2eq./yr for constant diet scenario on level of 1995, 15.3 Gt CO2eq./yr for increased meat scenario based on change in GDP and 9.78 Gt CO2eq./yr increased meat plus technological mitigation scenario) [18]. (PDF) [file pone.0062228.s004.pdf]

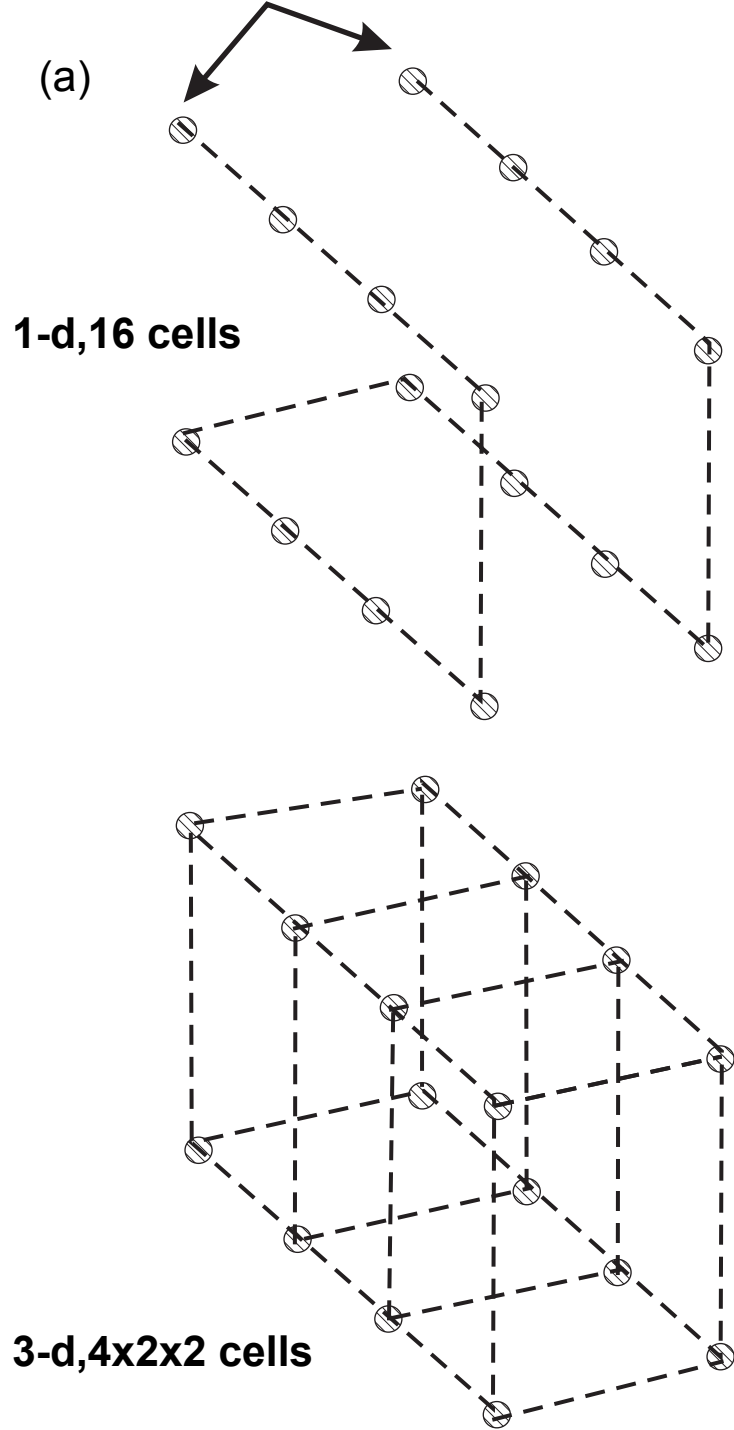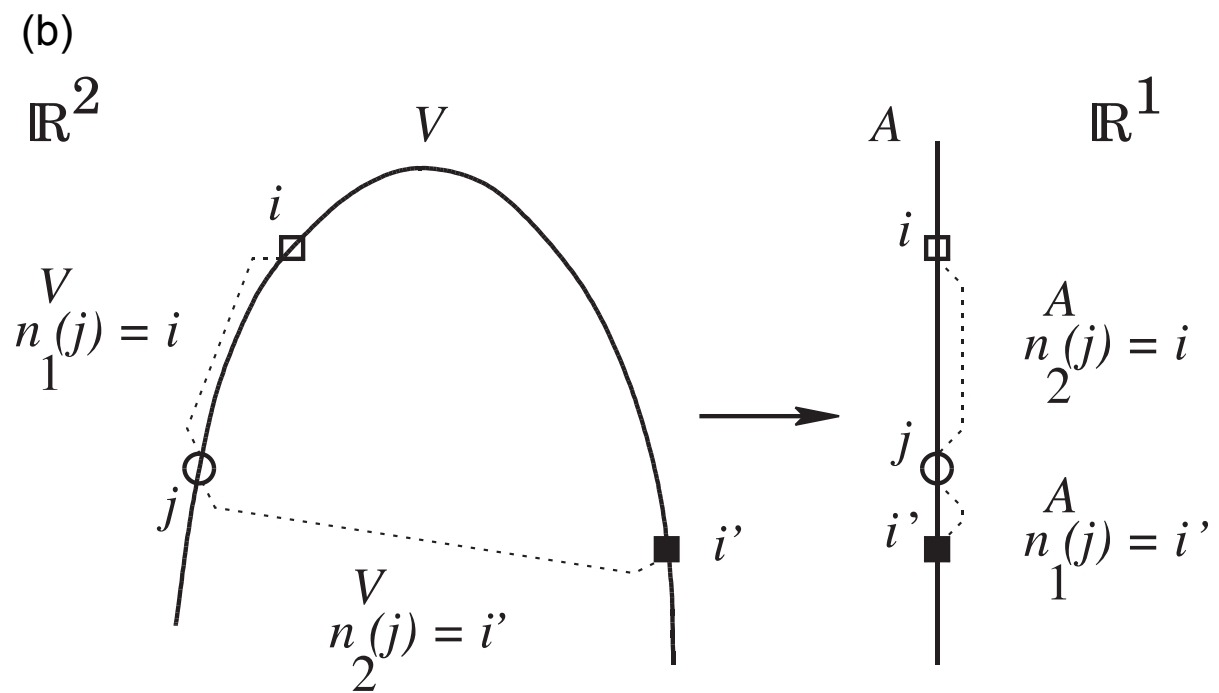

Supplement: Figure S5 — Schematic representation of a topology distortion.(a) Representation of a topology distortion between two different network types. Consider the bullets as scatter plots in . Such data distribution can be represented by a 1- with 16 cells or by 3- network with a geometry (16 cells). While for the 1- two bullets are direct neighbors in , while in there have a maximum distance (black arrows). Only the 3- network can represent this topological ordering adequately (cf. Table S2 for the results of the actual simulations). (b) Measurement of the distances from point to the next neighbors of order one and two, if the points lying in are mapped onto . It is shown how distance ratios can be used in order to quantify topological distortions. (PDF) [file pone.0062228.s005.pdf]
