# Supplementary material for: Embodied Greenhouse Gas Emissions in Diets
Source: PLoS One. 2013 May 15;8(5):e62228. doi: 10.1371/journal.pone.0062228 (PMC3655165; doi:10.1371/journal.pone.0062228)
Supplement: Table S1 — Number of observed diet transitions. The counts on the diagonal represent unchanged dietary patterns. Counts greater than 10 are used to plot the transition graph (Figure 4). Read from row to column, e.g. change recorded for Pattern 4 Pattern 5 is 27. (PDF) [file pone.0062228.s006.pdf]

Table S1. Number of observed diet transitions. The counts on the diagonal represent unchanged dietary patterns. Counts greater than 10 are used to plot the transition graph (Figure 4). Read from row to column, e.g. change recorded for Pattern 4 → Pattern 5 is 27.

Pattern	1	2	3	4	5	6	7	8	9	10	11	12	13	14	15	16
1	640	0	6	34	14	0	9	2	1	0	0	0	0	0	0	0
2	0	131	0	0	0	0	0	2	0	0	0	0	0	0	0	0
3	8	0	463	0	0	0	1	4	0	0	0	0	0	0	0	0
4	19	0	0	901	27	0	5	0	1	17	0	0	0	0	0	0
5	6	0	0	16	945	16	9	0	0	9	36	4	1	1	0	0
6	1	0	0	0	15	610	0	2	0	0	29	7	3	3	0	0
7	7	0	0	5	7	1	302	0	1	1	0	2	0	0	0	0
8	1	1	3	0	0	5	0	150	0	0	0	0	0	0	0	0
9	1	0	0	1	1	0	1	0	323	0	0	1	0	0	0	0
10	0	0	0	3	10	0	0	0	0	440	3	9	15	0	0	0
11	0	0	0	0	16	16	1	0	0	3	700	13	12	7	2	0
12	0	0	0	1	1	5	1	0	1	5	12	567	2	11	15	9
13	0	0	0	0	0	2	0	0	0	8	4	2	409	0	0	7
14	0	0	0	0	0	2	0	0	0	0	5	14	1	647	29	1
15	0	0	0	0	0	0	0	0	0	0	1	6	0	12	848	14
16	0	0	0	0	0	0	0	0	0	0	0	5	4	0	10	205
