# Supplementary material for: Embodied Greenhouse Gas Emissions in Diets
Source: PLoS One. 2013 May 15;8(5):e62228. doi: 10.1371/journal.pone.0062228 (PMC3655165; doi:10.1371/journal.pone.0062228)
Supplement: Table S2 — Topographical product and data reconstruction rates for SOM maps of certain dimensions.The map configuration with an optimal neighborhood preservation () indicated by a topographical product is used for further analysis (cf. arrow). It describes the 12-dimensional input manifold by a 3-dimensional embedding space and a data reconstruction rate of 70%. (PDF) [file pone.0062228.s007.pdf]

**Table S2.** Topographical product and data reconstruction rates for SOM maps of certain dimensions. The map configuration with an optimal neighborhood preservation ($4 \times 2 \times 2$) indicated by a topographical product $\equiv 0$ is used for further analysis (cf. arrow). It describes the 12-dimensional input manifold by a 3-dimensional embedding space and a data reconstruction of 70%.

| $d_{SOM}$ | Network geometry | Topographical product | Reconstruction Rate | |
|---|---|---|---|---|
| 1 | 8 | -0.022 ± 0.003 | 0.68 ± 0.0006 | |
| 2 | $4 \times 2$ | -0.014 ± 0.004 | 0.68 ± 0.0003 | |
| 1 | 10 | -0.019 ± 0.002 | 0.69 ± 0.0003 | |
| 2 | $5 \times 2$ | -0.015 ± 0.002 | 0.69 ± 0.0002 | |
| 1 | 12 | -0.013 ± 0.001 | 0.70 ± 0.0002 | |
| 2 | $4 \times 3$ | -0.014 ± 0.002 | 0.70 ± 0.0002 | |
| 2 | $6 \times 2$ | -0.023 ± 0.002 | 0.70 ± 0.0003 | |
| 3 | $3 \times 2 \times 2$ | -0.001 ± 0.002 | 0.70 ± 0.0002 | |
| 1 | 14 | -0.012 ± 0.001 | 0.71 ± 0.0003 | |
| 2 | $7 \times 2$ | -0.024 ± 0.002 | 0.71 ± 0.0002 | |
| 1 | 16 | -0.010 ± 0.001 | 0.71 ± 0.0002 | |
| 2 | $4 \times 4$ | -0.005 ± 0.001 | 0.72 ± 0.0003 | |
| 2 | $8 \times 2$ | -0.022 ± 0.002 | 0.72 ± 0.0002 | |
| 3 | $4 \times 2 \times 2$ | 0.002 ± 0.001 | 0.72 ± 0.0005 | ⟵ |
| 4 | $2 \times 2 \times 2 \times 2$ | 0.035 ± 0.003 | 0.72 ± 0.0003 | |
| 1 | 20 | -0.011 ± 0.001 | 0.71 ± 0.0004 | |
| 2 | $5 \times 4$ | -0.023 ± 0.002 | 0.73 ± 0.0003 | |
| 2 | $10 \times 2$ | -0.028 ± 0.003 | 0.73 ± 0.0002 | |
| 3 | $5 \times 2 \times 2$ | -0.015 ± 0.002 | 0.73 ± 0.0003 | |
| 1 | 24 | -0.015 ± 0.002 | 0.71 ± 0.0004 | |
| 2 | $6 \times 4$ | -0.028 ± 0.002 | 0.74 ± 0.0002 | |
| 2 | $12 \times 2$ | -0.020 ± 0.002 | 0.74 ± 0.0003 | |
| 3 | $4 \times 3 \times 2$ | -0.016 ± 0.002 | 0.74 ± 0.0002 | |
| 4 | $3 \times 2 \times 2 \times 2$ | 0.009 ± 0.001 | 0.74 ± 0.0003 | |
