# Supplementary material for: Embodied Greenhouse Gas Emissions in Diets
Source: PLoS One. 2013 May 15;8(5):e62228. doi: 10.1371/journal.pone.0062228 (PMC3655165; doi:10.1371/journal.pone.0062228)
Supplement: Text S1 — Characteristics of Identified Dietary Patterns. (PDF) [file pone.0062228.s008.pdf]

SUPPORTING INFORMATION

Embodied greenhouse gas emissions in diets

Prajal Pradhan[1*], Dominik E. Reusser[1], Juergen P. Kropp[1,2]

1 Potsdam Institute for Climate Impact Research, Potsdam, Germany

2 University of Potsdam, Dept. of Geo- and Environmental Sciences, Potsdam, Germany

∗ **E-mail: pradhan@pik-potsdam.de**

1 Characteristics of Identified Dietary Patterns

The derived sixteen dietary patterns are distinguished by their energy content and food composition. The diets are broadly categorized into low (<2,100 kcal/cap/day), moderate (2,100–2,400 kcal/cap/day), high (2,400–2,800 kcal/cap/day), and very high calorie diets (>2,800 kcal/cap/day) based on average energy requirements. An average of 2,100 kcal/cap/day is recommended as the minimum energy requirement for a typical population in a developing country, assuming a standard population distribution and body size, for survival and light physical activity [1]. For a moderately active lifestyle of a female population between age 18 to 30 with a mean height of 1.70 m, a mean energy intake of 2,400 kcal/cap/day is recommended [2]. For a male population with similar features the energy requirement is of 2,800 kcal/cap/day [2].

The characteristics of the dietary patterns are as follows:

1.1 Low Calorie Diets

Pattern #1 (avg. 1,870 kcal/cap/day) characterizes a food composition with cereals contributing to more than 50% of total energy supply. Developing countries in Africa, East Asia and small Island States are members of this pattern, e.g. Haiti (1984-1997), Madagascar (1988-2007), Timor-Leste (1961-2007), Botswana (1961-1978), Zambia (1993, 1998-2007), Burkina Faso (1961-1985), Chad (1965-2007), Eritrea (1993-2007), Ethiopia (1993-2007), Tanzania (1961-1967, 1969-2007), China (1961-1977), Indonesia (1961-1972).

Pattern #2 (avg. 2,000 kcal/cap/day) demonstrate a diet with a high fraction of pulses, fruits, starchy roots and alcoholic beverages as a basis for the food consumption pattern. It is also characterized by a low fraction of vegetables and vegetable oil in comparison to other patterns. Landlocked countries in Eastern Africa, e.g. as Rwanda, Uganda and Burundi were members of this dietary pattern for almost the entire period of the analysis.

Pattern #3 (avg. 2,018 kcal/cap/day) is characterized by the highest amount of starchy roots. Starchy roots, cereals and pulses contribute more than 70% of the total food supply. Mainly Middle African and West African countries like Angola, Benin, Congo, Cote d'Ivoire, Democratic Republic of Congo and Ghana belong to this class for the majority of years during 1961-2007.

1.2 Moderate Calorie Diets

Pattern #4 (avg. 2,140 kcal/cap/day) is a diet where cereals provide about 70% of the food energy supply. It prevailed mainly in Asian countries during their development phases, such as e.g. Azerbaijan (1992-2000), Saudi Arabia (1963-1970), Bangladesh (1961-2007), Cambodia (1961-2007), Indonesia (1973-2007), Vietnam (1961-2005), China (1978-1986), Republic of Korea (1961-1968), Tajikistan (1994-1995, 1998-2003). In addition, some developing African countries were also members, e.g. Kenya (1965-1979), Sudan (1992-1997), Burkina Faso (1986-2007), Mali (1970, 1985-2007), Chad (1961-1964), Lesotho (1961-2007), Zimbabwe (1961-1981).

For **pattern #5** (avg. 2,160 kcal/cap/day) the diet is composed of a very high amount of pulses, vegetable oil and sugar-sweetener and a very low amount of starchy roots. Pattern #5 is widely showing up among developing countries in Africa, South America, Central America, Asia and Small Island Nations. Some countries which belong to this pattern were: Morocco (1961-1982), Botswana (1979-2007), Namibia (1961-2007), Kenya (1980-1996, 1998-2007), Djibouti (1961-2005, 2007), Mauritania (1961-1974, 1976-1994), Suriname (1961-1979, 1981-1983, 1985-1990), Guatemala (1961-2007), Honduras (1961-1994), Mexico (1961-1972), Maldives (1961-1988, 1990), Pakistan (1962-2007), Iran (1961-1979), Malaysia (1961-1976), Philippines (1966-

2007), Mongolia (1963-1991, 1995-2007), Haiti (1998-2007), Seychelles (1961-1993, 2003-2006).

Pattern #6 (avg. 2,173 kcal/cap/day) exhibits the highest fraction of animal products and sugar-sweeteners. It was observed mainly in Central American, South American and Small Island countries, but for different time periods. Member countries of this pattern were: Brazil (1961-1973), Colombia (1961-1993), Costa Rica (1961-1972), Ecuador (1961-2007), Paraguay (1979-1984, 1986-1993), Dominica (1961-1978, 1980-1985), Saint Kitts and Nevis (1961-1973), Saint Lucia (1961-1998, 2001).

Pattern #7 (avg. 2,210 kcal/cap/day) is a diet characterized by the highest amount of vegetable oils and the lowest fraction of animal products in the current group. Mainly West African countries like Gambia, Guinea-Bissau, Liberia, Nigeria, Senegal and Sierra Leone were members of this pattern for almost the entire period (1961-2007).

The dietary **pattern #8** (avg. 2,270 kcal/cap/day) shows the highest value of sugar crops, a high value of fruits with cereal and starchy roots contributing 30% and 20% of total food energy supply, respectively. Two Central African countries, namely Cameroon and Gabon, belonged to the pattern for almost the entire period of 1961-2007, while two Western African countries, Cote d'Ivoire and Ghana were members for only a couple of years (1964-1972, 1975-1982 and 1975-1976, 2005-2007).

1.3 High Calorie Diets

Pattern #9 (avg. 2,540 kcal/cap/day) represents a diet with the highest consumption of starchy roots and fruits in this group and the overall highest supply of oil crops. Some of the Island states, Fiji (1961-1970), Kiribati (1961-2007), Samoa (1961-2007), Sao Tome and Principe (1961-1980, 1982-2007), Sri Lanka (1979-1980) and Vanuatu (1961-2007) are included in the pattern #9 diet.

Pattern #10 (avg. 2,580 kcal/cap/day) features the highest amount of cereals in the current group contributing more than 55% of total food supply. This pattern was found in some countries belonging to West Asia, North Africa, Former Soviet Union and in transition economies. Charac-

teristic countries were: Iran (1980-1989, 1993), Turkey (1961-1970), Egypt(1962-1981, 1983-2007), Tunisia (1971-1973, 1976-1978), Turkmenistan (1992-2007), Uzbekistan (1992-2007), China (1987-2007), Vietnam (2006-2007).

Pattern #11 (avg. 2,710 kcal/cap/day) is typical for a diet containing the highest portion of animal products, pulses, vegetable oils, sugar-sweetener, and alcoholic beverages among the high calorie diets. Some of the Small Island Nations belonged to pattern #11 diet for different time periods e.g. Dominica (1986-1990, 1994), French Polynesia (1962-1964), Saint Kitts and Nevis (1974-2007), Seychelles (1998-2001). After 1970 many countries in Central America and South America became members of this type, e.g. Belize (2005-2007), Costa Rica (1973-2007), Mexico (1973-2007), Brazil (1974-2007), Colombia (1994-2007), Uruguay (2007).

1.4 Very High Calorie Diets

For **pattern #12** (avg. 2,890 kcal/cap/day) animal products, cereals, vegetable oils and sugar-sweetener contributes around 20%, 40%, 10% and 10% of the total energy supply respectively. Countries in Central, South Eastern and Southern Europe, some in South America and Former Soviet Union are characteristic for this pattern. Countries which belonged to this pattern are: Slovakia (1993-1997, 1999-2007), Slovenia (1992-2007), Albania (1995-1996, 2007), Bosnia and Herzegovina (2005-2007), Romania (1967-2002, 2004, 2007), Italy (1961-1966), Portugal (1961-1986), Spain (1961-1972), Chile (1961-1985, 1988, 2004-2007), Guyana (1998-2003, 2005-2006), Paraguay (1985, 1994-2007), Belarus (1999-2005), Kazakhstan (1992-1997, 2000-2007), Russian Federation (1992-2006).

Pattern #13 (avg. 3,060 kcal/cap/day) exhibits the highest fraction of cereals pulses and vegetables and the lowest fraction of alcoholic beverages. Vegetal products contribute to more than 85% of the total food supply. It was mainly present in most of the countries in Western Asia and the Arab League during time periods after 1970. Characteristic countries of this pattern are: Algeria (1988-1997, 1999-2007), Iran (1990-1992, 1994-2007), Jordan (1994-1996, 2002-2007), Morocco (1986, 1988-2007), Saudi Arabia (1980-2007), Syria (1978-2005), Tunisia

(1974-1975, 1979-2007), Turkey (1971-2007), United Arab Emirates (1975-1979, 1982-2007).

Pattern #14 (avg. 3,110 kcal/cap/day) is a diet characterized by the overall highest amount of animal products and sugar-sweeteners, e.g. 35% of the energy supply comes from animal products. Mainly developed countries in Northern Europe, North America and Asia Pacific belonged to this pattern, such as Australia (1961-1979, 1981, 1984), Canada (1961-1979), Denmark (1961-1970, 1993-2007), Finland (1961-2000), Iceland (1961-2007), Ireland (1961-1984), New Zealand (1961-2007), Norway (1961-1994), United States of America (1961-1966).

Pattern #15 (avg. 3,300 kcal/cap/day) is qualified by the overall highest consumption of alcoholic beverages and exhibits the second highest amount of animal products and sugar-sweeteners. It is also mainly observed in developed countries in Northern Europe, North America and Asia Pacific, for example: Australia (1980, 1982-1983, 1985-2007), Austria (1963-2007), Canada (1980-1994), Finland (2001-2007), France (1961-2007), Germany (1964-2007), Ireland (1985-2007), Netherlands (1961-2007), Norway (1995-2007), Sweden (1966-2007), Switzerland (1961-2007), United States of America (1967-1999, 2003).

Finally **pattern #16** (avg. 3,430 kcal/cap/day) defines the class of countries with the highest total calorie intake. It is also associated with the highest consumption of vegetable oils, vegetables and fruits. The consumption of animal products is comparatively low in comparison to pattern #14 and #15, but higher than for pattern #12 and #13 diets. Developed countries in the Mediterranean region like Greece, Italy and Spain were members of this pattern mostly during the period from 1967 to 2007, whereas other developed countries shifted to this pattern later, e.g. like Canada (1995-2007), Belgium (2002-2005), USA (2000, 2002; 2004-2007).

References

1. UNHCR/UNICEF/WFP/WHO (2002) Food and Nutrition Needs in Emergencies. 51 pp.

2. FAO/WHO/UNU (2004) Human energy requirements. Rome. Italy, 103 pp.
