# Supplementary material for: Embodied Greenhouse Gas Emissions in Diets
Source: PLoS One. 2013 May 15;8(5):e62228. doi: 10.1371/journal.pone.0062228 (PMC3655165; doi:10.1371/journal.pone.0062228)
Supplement: Text S2 — Methods and Data. (PDF) [file pone.0062228.s009.pdf]

SUPPORTING INFORMATION

Embodied greenhouse gas emissions in diets

Prajal Pradhan[1*], Dominik E. Reusser[1], Juergen P. Kropp[1,2]

1 Potsdam Institute for Climate Impact Research, Potsdam, Germany

2 University of Potsdam, Dept. of Geo- and Environmental Sciences, Potsdam, Germany

∗ **E-mail: pradhan@pik-potsdam.de**

2 Methods and Data

2.1 The SOMTOP approach

Analysis and interpretation of large amounts of data has become one of the most important research tasks in earth systems science. Machine learning techniques such as artificial neural networks (ANNs) have several advantages in this regard. They are not only able to replicate the computational power of their biological examples but also are able to represent nonlinear relations, are capable of incorporating new information that is fed in, and are robust in handling noisy data. However, ANNs need large amounts of homogeneous data and the operator has to provide plausible explanations about why they approximate a solution. For the particular food data used in this study, the features of the ANN approach were explicitly wanted, i.e. certain patterns for diets should be derived and an optimal embedding, which allows the assessment of food transition pathways. For our analysis we implemented a neural network approach consisting of: i) a self-organizing map (SOM-) [1] and ii) an algorithm providing a quantitative measure P of topological distortions (-TOP) during the mapping of data on the neural network [2]. The employed model approach has been used for several other studies and has shown its effective performance in complex data analysis [3–6]. A SOM in particular can be interpreted as a clustering and non-linear dimensionality reduction technique. The SOM in addition tries to preserve the topological ordering of the input data in the low-dimensional network space. We

provide below a brief description of the applied approach. For a more detailed description of the algorithm, we refer to Kohonen and Bauer & Pawelzik [1,2].

2.1.1 Self-Organizing Maps

Kohonen's SOM is inspired by biological examples of neural networks: the brains of mammals. The neurons are organized in areas of the neocortex such that they reflect some physical characteristics of the signals stimulating them [7,8]. In a similar manner, a SOM extracts structural information from numerical data with an unsupervised learning process instead of memorizing all of it. During this process, an m-dimensional information continuum V is mapped onto an n-dimensional discrete space A, illustrating the structural information of the input data with a number of nodes (or neurons) corresponding to the dimension of the map, where normally $n < m$. Geometry of the discrete space can be either rectangular or hexagonal. Each node (i) is associated with a weight vector (ω) with the same dimension as of the input numerical data. In brief, Kohonen's algorithm can be formulated as follows:

1. Initialize the weight vectors (ω) for all nodes with random values
2. Iteratively, present a randomly chosen input vector (v) to the map, where all nodes compete to represent the input data with the following steps:
   (a) Compute the Euclidean distance between the input vector (ν) and all nodes, and select the output node (i) (best matching unit- BMU) that is most similar to ν;
   $$\parallel \nu - \omega_i \parallel \leq \ \min_{\forall j \in A} \parallel \nu - \omega_j \parallel,$$
   (b) Update the weight for the BMU and its neighbors according to the rule (learning process):
   $$\omega_i(t+1) = \omega_i(t) + \varepsilon(t) \times h_{i,j} \times [\nu(t) - \omega_i(t)]$$
   where $\varepsilon(t)$ is a learning parameter that decreases to zero with iteration, $h_{i,j}$ is a time-dependent neighborhood function - often a Gaussian function, that defines the

vicinity of the mesh in which other nodes learn from the same input stimulus

(c) Calculate the average change rate of the map or the mapping error and stop the learning process if it is less than a predefined threshold value

Artificial neural networks are adaptive models that change their structure during learning processes. They generalize the things learned from data and visualize the non-linear relations of multidimensional data. The data are finally represented by a hyperplane of lower dimensionality, which is embedded within the data space. This technique offers a convenient method to reduce the amount of information as well as to form an implicit model, without having to form a traditional physical model of the underlying problem.

2.1.2 Topological Ordering

During the learning process, the aggregation of similar objects onto a neuron is a topology preserving representation of the input data. The mapping divides the input space in a type of Voronoi segmentation [9]. Each node represents one of the gravity centers of the partially segmented input information continuum V. Therefore, it is not possible to describe the data in a simple way and with optimal quality if the dimensionality of the input data and the network differ [10]. Additionally, unsuitable chosen training parameters may entail topologically distorted mappings (cf. Figure S5a). Therefore, a measure to estimate the quality of the mapping is needed. This can be provided by the calculation of the topographical product (P) [2]. It provides a measure of topology distortions in maps between spaces of possibly different dimensionality. According to Bauer & Pawelzik [2] two distance ratios firstly have to be defined:

$$Q_1(j,k) = \frac{D^V\big(\omega_j, \omega_{n_k^A(j)}\big)}{D^V\big(\omega_j, \omega_{n_k^V(j)}\big)} \tag{1}$$

and

$$Q_2(j,k) = \frac{D^A\big(j, n_k^A(j)\big)}{D^A\big(j, n_k^V(j)\big)} \tag{2}$$

In equations 1 and 2, $n_k^V(j)$ and $n_k^A(j)$ denote the k-th order (next) neighbor of the point j in the input and output space, respectively (cf. Figure S5b). At first, the distance between the points is measured in the input space (D^V) and output space (D^A) by using the node coordinates j and the weight vectors (ω_j). Figure S5b illustrates the neighbors of j. In the $\mathbb{R}^2$, the neighbor is given by $n_1^V(j) = i$ and in the $\mathbb{R}^1$ by $n_1^A(j) = i'$. For the distance ratio measured in the input space V, we obtain $Q_1(j,1) > 1$, because $D^V(j,i') > D^V(j,i)$. However, in the output space A, we get $Q_2(j,1) < 1$. This indicates the neighborhood distortion in the mapping from $\mathbb{R}^2$ to $\mathbb{R}^1$. Only when $Q_1 = Q_2 = 1$, the points in A and V coincide and the topology is preserved. Thus, P measures the preservation of the neighborhood between the neural units i in A and their weight vectors ω_i lying on V. It is defined as follow:

$$P = \frac{1}{N(N-1)} \left(\sum_{j=1}^{N} \sum_{k=1}^{N-1} \log \left[\prod_{l=1}^{k} Q_1(j,l)\ Q_2(j,l) \right]^{\frac{1}{2k}} \right) \tag{3}$$

When the preservation of the neighborhood relations is achieved, P equals zero. $P > 0$ and $P < 0$ indicate dimensions that are too large or too small, respectively. Due to the case that this is an approximation process, a absolute zero is in most cases not achievable (cf. Table S2 for the current simulations). An additional precondition in minimizing $|P|$ is to adjust the learning parameters. Only in this case, the error caused by the random training process can be minimized as well and therewith the network converges to a topographic map [11].

Our approach makes use of the features of both analytical concepts, because food production and food consumption have neighborhood relations in terms of its geographical distribution. Nevertheless, the topological properties can be distorted during this mapping process, due to i) an unsuitable selection of the dimension of the output space or ii) inappropriate training parameters. For concrete classification and identification of transitions, the topological ordering is of additional interest because certain transition trajectories have a topological relationship to similar time developments in the original data space. Consequently an erroneous mapping may lead to false interpretations. An optimal embedding space (network dimension) was found

when $P \approx 0$ (cf. Table S2). Due to the stochastic nature of the learning process, the challenge is to approximate P adequately. First the dimension for the SOMTOP simulations needs to be identified. For this question, we employ as a linear embedding approach, the principal component analysis (PCA) [12]. The PCA yields $d_{PCA} = 4$, i.e. 4 Eigenvalues $\gamma = 4.05, 1.98, 1.22, 1.08$ larger than 1, explaining 70% of the variance. Assuming that complex input data have a nonlinear nature the $d_{PCA} = 4$ can be used as the upper constraint for our simulations, i.e. the SOMTOP simulations were performed for a one- to four-dimensional output data space. (cf. Table S2). These simulations provided that the best representation of the input data is guaranteed by a $4 \times 2 \times 2$ network configuration explaining a variance of 72%. Consequently, we use this result for further detailed analysis.

2.2 Data Sources and processing

The input data set consists of 9 145 data sets comprising 12 input variables (animal products, cereals, pulses, starchy roots, oil crops, vegetable oils, vegetables, fruits, sugar and sweeteners, sugar crops and alcoholic beverages, and total food consumption) for 217 countries and country groups e.g. Asia, Europe, World, etc. covering a time period from 1961-2007. The different food groups account for more than 90% of the global food supply and are measured in kcal/capita/day (cf. Food Balance Sheets of FAO, e.g. [13, 14]). The data provide numbers regarding food availability not on actual consumption and do not cover losses which may happen due to the refining of food during the food production chain. This may limit the usability of the data, but the existing data are the most comprehensive and consequently we use them as a proxy for food consumption.

Nutritive factor data contains a conversion factor for converting the amount of crop and animal products provided from grams to calories [13]. To estimate necessary feed for livestock in kcal/cap/day (F) per country, we consider the total crop amount used as fodder by converting its supply in tons/yr into kcal/yr using the nutritive factors of crops and dividing them by the country population and by 365 in order to calculate a daily value.

As recently indicated, there exists a linear relationship between the HDI (Human Development Index) [15], which measures the development level (GDP per capita, life expectancy, enrollment rate, etc.) and log CO_2 emissions per cap [16]. Due to the fact that the HDI can be considered as an indirect proxy for life style changes, it was used to project food consumption pattern and their embodied emissions. To estimate the HDI related to dietary patterns, we employed data on HDI trends 1980-2007 from the Human Development Report 2009. The data are available starting from the year 1980 in 5 years' time intervals.

The estimation of fossil energy and the related GHG emissions embodied in certain dietary patterns was performed on energy output/input (O/I) ratio ($R_{I/O}$) data obtained from Conforti and Giampietro [17], who estimated energy O/I ratio for agricultural products for an average of 1990-1991 for 66 countries. The energy O/I ratio is defined as the ratio between food energy obtained from agricultural products and the fossil energy necessary for its production.

For the estimation of non-CO_2 GHG emissions, data from US-EPA [18] and data on crops and livestock production from FAOSTAT were utilized [14]. Using respective nutritive factors for crops and livestock items we converted total crops and livestock production from tons to calorific values. The non-CO_2 emissions from agriculture consist of GHG emissions from enteric fermentation, rice cultivation, manure management and agricultural soils. The emissions data was split into crop related (rice and soils) and livestock related (enteric fermentation and manure management) emissions. We calculated non-CO_2 GHG emission intensity per kcal of crop products (ec) and animal products (ea) for each country by dividing the crop and livestock production data in caloric values with the crop and livestock related non-CO_2 GHG emissions data.

Results obtained from SOMTOP simulations provide sixteen diet typologies, each of them representing a set of country and year pairs (Z) characterized by a certain food composition and total food consumption feature. Considering the set (Z) and the X' as a set of pairs of country (C) and year (Y) for which data on X (energy O/I ratio, non-CO_2 GHG emission intensity and feed use) is available, the average value (X_z) related to the dietary pattern was obtained by

equation (4).

$$X_z = \frac{1}{\#(Z \cap X')} \sum_{(C,Y) \in Z \cap X'} X(C,Y) \tag{4}$$

The total GHG emissions (ET_z) embedded in a dietary pattern was divided into GHG emissions from crops (EC_z) and livestock (EA_z) based on consumption of crop products (PC_z) (total food consumption minus animal products consumption) and animal products (PA_z). Considering crops as major livestock feed, we calculated additional non-CO_2 and fossil emissions embodied in livestock products. The GHG emissions from fossil energy was estimated using the emission intensity of diesel (eD), which is 0.36 g $CO_{2\mathrm{eq.}}$ per kcal [19]. Applying equations (5), (6) and (7), we calculated GHG emissions from crop products, animal products and total food consumption related to a specific dietary pattern, respectively and with equation (8) we calculated the embedded fossil energy (FE_z).

$$EC_z = ec_z \times PC_z + R_{O/Iz} \times PC_z \times eD \tag{5}$$

$$EA_z = ea_z \times PA_z + R_{O/Iz} \times F_z \times eD + ec_z \times F_z \tag{6}$$

$$ET_z = EC_z + EA_z \tag{7}$$

$$FE_z = R_{O/Iz} \times PC_z + R_{O/Iz} \times F_z \tag{8}$$

References

1. Kohonen T (2001) Self-Organizing Maps. Springer Series in Informations Sciences. Berlin: Springer, 501 pp.

2. Bauer HU, Pawelzik KD (1992) Quantifying the neighborhood preservation of self-organizing feature maps. IEEE Trans Neural Netw 3: 570–579.

3. Ambroise C, Sèze G, Badran F, Thiria S (2000) Hierarchical clustering of self-organizing maps for cloud classification. Neurocomputing 30: 47-52.

4. Crane RG, Hewitson BC (2003) Clustering and upscaling of station precipitation records to regional patterns using self-organizing maps (SOMs). Climate Research 25: 95-107.

5. Hanewinkel M, Zhou W, Schill C (2004) A neural network approach to identify forest stands suceptible to wind damage. Forest Ecology and Management 196: 227-243.

6. Kropp JP, Schellnhuber HJ (2008) Prototyping Broad-Scale Climate and Ecosystem Classes by Means of Self-Organising Maps, Chichester: Wiley, chapter 9. pp. 155–175.

7. Bauer DR H U, Herrmann M (1996) Controlling the magnification factor of self-organizing feature maps. Neural Comput 8: 757–771.

8. Bauer GTPK H U, Wolf F (1996) Selbstorganisierende neuronale karten. Spektrum der Wissenschaft 4: 38–47.

9. Voronoi G (1908) Nouvelles applications des paramètres continus à la théorie des formes quadratiques. Deuxième mémoire. Recherches sur les parallélloèdres primitifs. Journal für die reine und angewandte Mathematik (Crelles Journal) : 198–287.

10. Li X, Gasteiger J, Zupan J (1993) On the topology distortion in self-organizing feature maps. Biological Cybernetics 70.

11. Ritter H, Schulten K (1988) Convergence properties of kohonen's topology conserving maps: fluctuations, stability, and dimension selection. Biological Cybernetics 60: 59-71.

12. Jolliffe I (2002) Principal Component Analysis, 2nd Edition. New York: Springer-Verlag.

13. FAO (2001) Food balance sheets: A handbook. Rome: Food and Agriculture Organization, 99 pp.

14. FAO (2011) FAOSTAT 2011, FAO Statistical Databases: Agriculture, Fisheries, Forestry, Nutrition. Rome: Food and Agriculture Organization of the United Nations.

15. UNDP (2009) Human Development Report 2009: Overcoming barriers: Human mobility and development. New York: UNDP.

16. Costa L, Rybski D, Kropp JP (2011) A human development framework for CO_2 reductions. PLoS ONE 6: e29262.

17. Conforti P, Giampietro M (1997) Fossil energy use in agriculture: an international comparison. Agric Ecosyst Environ 65: 231–243.

18. US-EPA (2006) Global Anthropogenic Non-CO_2 Greenhouse Gas Emissions:1990-2020. United States Environmental Protection Agency.

19. DFT-UK (2008) Carbon and Sustainability Reporting Within the Renewable Transport Fuel Obligation. Department for Transport.
