# Supplementary material for: Embodied Greenhouse Gas Emissions in Diets
Source: PLoS One. 2013 May 15;8(5):e62228. doi: 10.1371/journal.pone.0062228 (PMC3655165; doi:10.1371/journal.pone.0062228)
Supplement: Text S3 — Scenario Assumptions for the Estimation of GHGs from Agriculture. (PDF) [file pone.0062228.s010.pdf]

SUPPORTING INFORMATION

Embodied greenhouse gas emissions in diets

Prajal Pradhan[1*], Dominik E. Reusser[1], Juergen P. Kropp[1,2]

1 Potsdam Institute for Climate Impact Research, Potsdam, Germany

2 University of Potsdam, Dept. of Geo- and Environmental Sciences, Potsdam, Germany

∗ **E-mail: pradhan@pik-potsdam.de**

3 Scenario Assumptions for the Estimation of GHGs from Agriculture

For the estimation of future GHG emissions from agriculture, three different scenarios were defined and the following factors were considered: i) population change, ii) changes in food consumption, iii) non-CO_2 GHG emissions from crops and livestock, and iv) O/I ratio (cf Text S2). For the population change, data representing the mid-range population scenario for 148 countries was considered from UN 2011 [1]. The estimation of future food consumption was based on a relationship between the Human Development Index (HDI) and calorie intake. Exponential relationships were found between the amount of total calories, animal products, sugar-sweeteners, vegetable oils and vegetables consumption and the HDI values (Figure 3). Using the HDI projections from Costa et al. [2] and the exponential relationships between HDI and calories intake, we forecasted the food consumption and identified the dietary patterns the countries will belong to. The pattern with the least euclidian distance to the projected food consumption of a country was considered to be the future dietary pattern of the country. Changes in the non-CO_2 GHG emission intensities (crop and livestock) and the O/I ratio (cf. Table 1 and Text S2) for the countries were estimated, according to changes in the certain dietary pattern of a country. The scenarios analysis was performed until 2050 for time steps of 5 years. Three scenarios were analyzed.

3.1 Scenario A

The countries will not change their dietary pattern (2007) for the entire period of analysis. Furthermore, the non-CO_2 GHG emission intensities (crop and livestock) and the O/I ratio of the countries remain the same as the values associated with their dietary pattern of 2007. Only population changes were considered.

3.2 Scenario B

Food consumption changes, which are related to projected HDI changes for the countries, were accounted for. The non-CO_2 GHG emission intensities and the O/I ratio of the countries remain the same as in scenario A. The population changes as in scenario A.

3.3 Scenario C

Population and food consumption change were accounted for along with non-CO_2 GHG emission intensities and the O/I ratio for the countries vary over time. Thus, this scenario not only takes into account dietary transitions, but also assumes that the countries will adapt non-CO_2 GHG efficient agricultural technologies as observed historically for countries with the corresponding dietary pattern.

References

1. United Nations (2011) The Millennium Development Goals Report 2011 : 67.

2. Costa L, Rybski D, Kropp JP (2011) A human development framework for CO_2 reductions. PLoS ONE 6: e29262.
