# Supplementary material for: Correction: Embodied Greenhouse Gas Emissions in Diets
Source: PLoS One. 2016 Jul 8;11(7):e0159285. doi: 10.1371/journal.pone.0159285 (PMC4938127; doi:10.1371/journal.pone.0159285)
Supplement: S2 Text — (DOCX) [file pone.0159285.s001.docx]

Text S2. Methods and Data.

## 2.2 Data Sources and processing

The input data set consists of 9,145 data sets comprising 12 input variables (animal products, cereals, pulses, starchy roots, oil crops, vegetable oils, vegetables, fruits, sugar and sweeteners, sugar crops and alcoholic beverages, and total food consumption) for 217 countries and country groups e.g. Asia, Europe, World, etc. covering a time period from 1961-2007. The different food groups account for more than 90% of the global food supply and are measured in kcal/capita/day (cf. Food Balance Sheets of FAO, e.g. [13, 14]). The data provide numbers regarding food availability not on actual consumption and do not cover losses which may happen due to the refining of food during the food production chain. This may limit the usability of the data, but the existing data are the most comprehensive and consequently we use them as a proxy for food consumption.

Nutritive factor data contains a conversion factor for converting the amount of crop and animal products provided from grams to calories [13]. To estimate necessary feed for livestock in kcal/cap/day (*F*) per country, we consider the total crop amount used as fodder by converting its supply in tons/yr into kcal/yr using the nutritive factors of crops and dividing them by the country population and by 365 in order to calculate a daily value.

As recently indicated, there exists a linear relationship between the HDI (Human Development Index) [15], which measures the development level (GDP per capita, life expectancy, enrollment rate, etc.) and log CO_2_ emissions per cap [16]. Due to the fact that the HDI can be considered as an indirect proxy for life style changes, it was used to project food consumption pattern and their embodied emissions. To estimate the HDI related to dietary patterns, we employed data on HDI trends 1980-2007 from the Human Development Report 2009. The data are available starting from the year 1980 in 5 years’ time intervals.

The estimation of fossil energy and the related GHG emissions embodied in certain dietary patterns was performed on energy output/input (O/I) ratio ($R_{O/I}$) data obtained from Conforti and Giampietro [17], who estimated energy O/I ratio for agricultural products for an average of 1990-1991 for 66 countries. The energy O/I ratio is defined as the ratio between food energy obtained from agricultural products and the fossil energy necessary for its production.

For the estimation of non-CO_2_ GHG emissions, data from US-EPA [18] and data on crops and livestock production from FAOSTAT were utilized [14]. Using respective nutritive factors for crops and livestock items we converted total crops and livestock production from tons to calorific values. The non-CO_2_ emissions from agriculture consist of GHG emissions from enteric fermentation, rice cultivation, manure management and agricultural soils. The emissions data was split into crop related (rice and soils) and livestock related (enteric fermentation and manure management) emissions. We calculated non-CO_2_ GHG emission intensity per kcal of crop products (*ec*) and animal products (*ea*) for each country by dividing the crop and livestock production data in caloric values with the crop and livestock related non-CO_2_ GHG emissions data.

Results obtained from SOMTOP simulations provide sixteen diet typologies, each of them representing a set of country and year pairs (*Z*) characterized by a certain food composition and total food consumption feature. Considering the set (*Z*) and the *X´* as a set of pairs of country (*C*) and year (*Y*) for which data on *X* (energy O/I ratio, non-CO_2_ GHG emission intensity and feed use) is available, the average value ($X_{z}$) related to the dietary pattern was obtained by equation 4.

$X_{z}=\frac{1}{\#(Z\cap X')}\sum_{(C,Y)\in Z\cap X'} X(C,Y)$ (4)

The total GHG emissions (${ET}_{z}$) embedded in a dietary pattern was divided into GHG emissions from crops (${EC}_{z}$) and livestock (${EA}_{z}$) based on consumption of crop products (${PC}_{z}$) (total food consumption minus animal products consumption) and animal products (${PA}_{z}$). Considering crops as major livestock feed, we calculated additional non-CO_2_ and fossil emissions embodied in livestock products. The GHG emissions from fossil energy was estimated using the emission intensity of diesel (*eD*), which is 0.36 g CO_2eq._ per kcal [19]. Applying equations (5), (6) and (7), we calculated GHG emissions from crop products, animal products and total food consumption related to a specific dietary pattern, respectively and with equation (8) we calculated the embedded fossil energy (${FE}_{z}$).

${EC}_{z}={ec}_{z}\times{PC}_{z}+ \frac{{PC}_{z}}{R_{O/I_{z}}}\times eD$ (5)

${EA}_{z}={ea}_{z}\times{PA}_{z}+ \frac{F_{z}}{R_{O/I_{z}}}\times eD+{ec}_{z}\times F_{z}$ (6)

${ET}_{z}={EC}_{z}+{EA}_{z}$ (7)

${FE}_{z}= \frac{{PC}_{z}+ F_{z}}{R_{O/I_{z}}}$ (8)

## Reference

1. Pradhan P, Reusser DE, Kropp JP (2013) Embodied Greenhouse Gas Emissions in Diets. PLoS ONE 8(5): e62228. doi:10.1371/journal.pone.0062228
